# Treatments targeting autophagy ameliorate the age-related macular degeneration phenotype in mice lacking APOE (apolipoprotein E)

**DOI:** 10.1080/15548627.2022.2034131

**Published:** 2022-02-23

**Authors:** Kirstan A Vessey, Andrew I Jobling, Mai X. Tran, Anna Y. Wang, Ursula Greferath, Erica L. Fletcher

**Affiliations:** Department of Anatomy and Physiology, The University of Melbourne, Level 5, Medical Building, Grattan St, Parkville, Victoria, Australia

**Keywords:** B6.129P2-Apoe^*tm1Unc*^J, bruch’s membrane, metformin, retina, retinal pigment epithelium, trehalose

## Abstract

Age-related macular degeneration (AMD) is a leading cause of vision loss with recent evidence indicating an important role for macroautophagy/autophagy in disease progression. In this study we investigate the efficacy of targeting autophagy for slowing dysfunction in a mouse model with features of early AMD. Mice lacking APOE (apolipoprotein E; B6.129P2-Apoe^*tm1Unc*^J/Arc) and C57BL/6 J- (wild-type, WT) mice were treated with metformin or trehalose in the drinking water from 5 months of age and the ocular phenotype investigated at 13 months. Control mice received normal drinking water. APOE-control mice had reduced retinal function and thickening of Bruch’s membrane consistent with an early AMD phenotype. Immunohistochemical labeling showed reductions in MAP1LC3B/LC3 (microtubule-associated protein 1 light chain 3 beta) and LAMP1 (lysosomal-associated membrane protein 1) labeling in the photoreceptors and retinal pigment epithelium (RPE). This correlated with increased LC3-II:LC3-I ratio and alterations in protein expression in multiple autophagy pathways measured by reverse phase protein array, suggesting autophagy was slowed. Treatment of APOE-mice with metformin or trehalose ameliorated the loss of retinal function and reduced Bruch’s membrane thickening, enhancing LC3 and LAMP1 labeling in the ocular tissues and restoring LC3-II:LC3-I ratio to WT levels. Protein analysis indicated that both treatments boost ATM-AMPK driven autophagy. Additionally, trehalose increased p-MAPK14/p38 to enhance autophagy. Our study shows that treatments targeting pathways to enhance autophagy have the potential for treating early AMD and provide support for the use of metformin, which has been found to reduce the risk of AMD development in human patients.**Abbreviations:**AMD: age-related macular degeneration; AMPK: 5’ adenosine monophosphate-activated protein kinase APOE: apolipoprotein E; ATM: ataxia telangiectasia mutated; BCL2L1/Bcl-xL: BCL2-like 1; DAPI: 4ʹ-6-diamidino-2-phenylindole; ERG: electroretinogram; GAPDH: glyceraldehyde-3-phosphate dehydrogenase; GCL: ganglion cell layer; INL: inner nuclear layer; IPL: inner plexiform layer; IS/OS: inner and outer photoreceptor segments; LAMP1: lysosomal-associated membrane protein 1; MAP1LC3B/LC3: microtubule-associated protein 1 light chain 3 beta; MTOR: mechanistic target of rapamycin kinase; OCT: optical coherence tomography; ONL: outer nuclear layer; OPs: oscillatory potentials; p-EIF4EBP1: phosphorylated eukaryotic translation initiation factor 4E binding protein 1; p-MAPK14/p38: phosphorylated mitogen-activated protein kinase 14; RPE: retinal pigment epithelium; RPS6KB/p70 S6 kinase: ribosomal protein S6 kinase; SQSTM1/p62: sequestosome 1; TP53/TRP53/p53: tumor related protein 53; TSC2: TSC complex subunit 2; WT: wild type

## Introduction

Age-related macular degeneration (AMD) is a disease where progressive and irreversible vision loss occurs. It is initially characterized by the formation of extracellular deposits, called drusen, that accumulate in the posterior of the eye between the retinal pigment epithelium (RPE) and Bruch’s membrane [1,2]. Additionally, intracellular waste called lipofuscin accumulates within the RPE [1,3] and Bruch’s membrane thickens with lipid rich debris, limiting nutrient and oxygen transfer from the ocular blood supply to the neural retina [4–6]. This is followed by death of cells that are critical for vision; the light sensitive photoreceptors and the cells that support them, the RPE. There are two clinically distinct forms of end stage disease, neovascular and atrophic AMD [1,2]. While there are treatments that slow vision loss in neovascular AMD, there are no therapies to slow progression from early to late AMD or the continued development of atrophic AMD. There are common changes in the early stages of AMD that if detected and treated may slow progression to irreversible vision loss.

Recently, a range of studies have suggested that intracellular waste turnover and metabolism pathways are deregulated in AMD [7–9]. With age, waste build up has been suggested to overwhelm the normal process of autophagy, whereby degradation of macromolecules and organelles, such as mitochondria, no longer occurs effectively [10,11]. In most cells, autophagy occurs at basal levels to remove damaged organelles as part of a cellular repair process and also to provide nutrients for the cell in times of metabolic insufficiency. There is increasing evidence suggesting a role for autophagic impairment in a wide range of age-related neurodegenerative diseases, including AMD [7,8]. This highlights the possibility that treatments that enhance autophagy may limit AMD pathology. In particular, trehalose, a non-reducing disaccharide, and metformin, an agent used commonly to treat type II diabetes, show promise. Both are known to enhance autophagy by activating pathways within the cell which would normally occur during times of metabolic insufficiency, for example during caloric restriction, activating mechanisms that enhance macro-autophagy [9,12,13]. Supporting their potential benefit in AMD, these drugs have been shown to reduce lipofuscin accumulation and extend lifespan of the animal in *C. elegans* and mice [14–16]. Furthermore, metformin has recently been shown to reduce the risk of AMD development in humans [17]. In this study we will investigate the potential of drugs targeting autophagy as therapies for slowing dysfunction in a mouse model with features of early AMD.

In humans, AMD has been linked to mutations in several cholesterol-related genes including *APOE, LIPC, CETP*, and *ABCA1* [2,18] and inheritance of the ɛ2 APOE (apolipoprotein E) allele confers an increased risk of developing the disease [19]. In line with this, mice lacking APOE (APOE-mice) may provide a useful model of early AMD [18,20–22]. APOE is important for the transport of lipid across cell membranes and is highly expressed by the RPE [23]. Mice lacking APOE exhibit altered lipid and sugar metabolism [24] and develop thickened Bruch’s membrane, as well as accumulation of lipid deposits in the basal RPE and Bruch’s membrane [18,22]. In the current study we investigated whether treatments targeting autophagy modulate the ocular phenotype of APOE-mice and assess changes in autophagy pathways in retinal neurons and the RPE. APOE-mice and C57BL/6 J- (wild-type, WT) mice were treated with metformin or trehalose in the drinking water from 5 months of age and the ocular phenotype investigated at 13 months. Normal drinking water was provided for control animals. APOE-control mice had ocular changes consistent with an early AMD phenotype which correlated with alterations in protein expression in multiple autophagy pathways in both the photoreceptors and RPE. Treatment of APOE-mice with metformin or trehalose ameliorated the loss of retinal function and reduced Bruch’s membrane thickening, enhancing LC3 and LAMP1 labeling in the ocular tissues and restoring LC3-II:LC3-I ratio to WT levels. Both treatments were found to enhance the ATM-AMPK-driven autophagy pathway. Our study shows treatments targeting pathways that regulate metabolism and autophagy may be worth evaluating further for their potential to slow the progression of AMD.

## Results

### APOE-mice show retinal functional deficits at 13 months, which are ameliorated by treatment with trehalose or metformin

**Figure 1.**
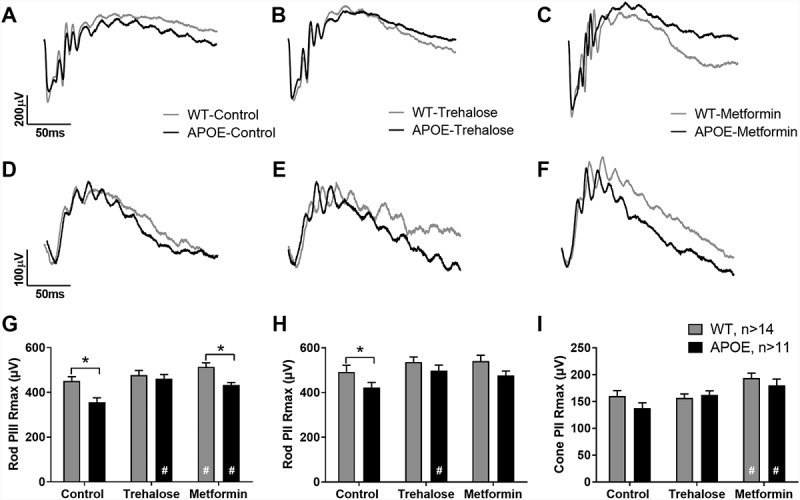
APOE-mice show retinal functional deficits at 13 months, which are ameliorated by treatment with trehalose or metformin. WT- and APOE-mice were treated from 5 months of age until 13 months with metformin (0.4 g/kg/day) or trehalose (3 g/kg/day) and retinal function was assessed using twin flash electroretinogram. (A-C) Rod pathway responses are presented for (A) untreated controls, (B) trehalose-treated, and (C) metformin-treated WT (gray line) and APOE mice (black line). (D-F) Cone pathway responses are presented for (D) untreated controls, (E) trehalose-treated, and (F) metformin-treated WT (gray line) and APOE mice (black line). (G) APOE mice (black bars) had a reduced rod photoreceptor response (Rod PIII Rmax, a-wave) and (H) post-photoreceptor response (Rod PII Rmax, b-wave) relative to WT-control mice (gray bars) and this loss was no longer apparent following treatment. (I) While there was no apparent cone pathway deficit, metformin treatment enhanced cone post-photoreceptor responses in WT- and APOE-mice relative to their genetic controls. For all groups n ≥ 11; Two way ANOVA with post-hoc significance of p < 0.05 shown for genotype (*) and treatment (#).

WT- and APOE-mice were treated from 5 months of age until 13 months with the autophagy enhancers metformin (0.4 g/kg/day) or trehalose (3 g/kg/day) provided in the drinking water. At 13 months, retinal function was assessed in all cohorts of animals (n ≥ 11 for all) using the electroretinogram (Figure 1). Representative traces of rod and cone pathway responses are presented for untreated WT-control and APOE-control mice (Figure 1A,D respectively), trehalose treated WT- and APOE-mice (Figure 1B,E respectively) and metformin treated WT- and APOE-mice (Figure 1C,F respectively). Rod response waveform analysis showed that mice lacking APOE had a reduced rod photoreceptor response (Rod PIII Rmax, a-wave) and post-photoreceptor response (Rod PII Rmax, b-wave) relative to WT-control mice (Figure 1G,H respectively; Two way ANOVA with significance of p < 0.05 for genotype indicated by * and treatment by #). Treatment of APOE-mice with trehalose or metformin was found to ameliorate this loss of rod photoreceptor function compared with APOE-controls (Figure 1G; Two way ANOVA with significance of p < 0.05 for treatment indicated by #). However, APOE-metformin treated animals had reduced photoreceptor response relative to WT-metformin treated animals (Figure 1G; p < 0.05 for treatment indicated by *), predominantly because metformin treatment was found to enhance rod photoreceptor function in WT-mice compared with WT-controls, suggesting slowing of retinal degeneration with age (p < 0.05 for treatment indicated by #). While there was no cone pathway deficit observed in any cohort, metformin treatment enhanced cone post-photoreceptor responses in WT- and APOE-mice relative to their genetic controls (Figure 1I; Two way ANOVA with significance of p < 0.05 for treatment indicated by #).

### Retinal functional changes in APOE- and drug treated-animals correlates with Bruch’s membrane thickness changes

**Figure 2.**
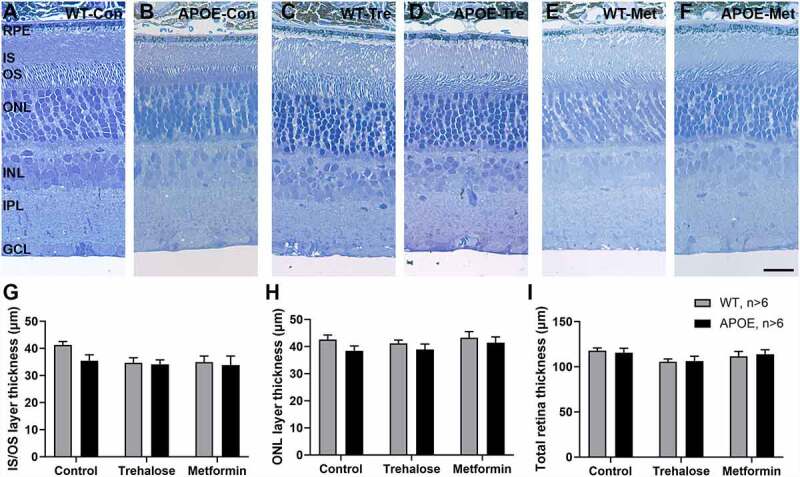
Retinal layer thickness is not altered in APOE-mice or as a result of drug treatment. Retinal morphology and layer thickness was assessed using semi-thin resin sections stained with toluidine blue. (A-F) Representative transverse retinal sections from (A) WT-control, (B) APOE-control, (C) WT-mice treated with trehalose, (D) APOE-mice treated with Trehalose, (E) WT-mice treated with metformin and (F) APOE-mice treated with metformin are presented. (G-I) There were no significant changes in (G) photoreceptor inner segment/outer segment length, (H) the outer nuclear layer or (I) total retinal thickness between WT- and APOE-mice and drug treated animals. For all groups n = 6; Two way ANOVA for genotype and treatment, p > 0.05. Scale: 20 µm. RPE, retinal pigment epithelium; OS, photoreceptor outer segments; IS, photoreceptor inner segments; ONL, outer nuclear layer; INL, inner nuclear layer; IPL, inner plexiform layer; GCL, ganglion cell layer.

Retinal functional change has been found to correlate with retinal fundus changes, loss of cells in the retinal layers and also Bruch’s membrane thickness [25]. Imaging of the retinal fundus and retinal layers by optical coherence tomograph (OCT) and subsequent analysis of retinal layer thickness showed there were no changes in APOE-mice relative to WT-controls or in animals treated with metformin or trehalose (Figure S1, Table 1). Detailed analysis of retinal morphology using semi-thin resin sections stained with toluidine blue was also undertaken (Figure 2). Representative transverse retinal sections from WT-control (Figure 2A), APOE-control (Figure 2B), WT-mice treated with trehalose (Figure 2C), APOE-mice treated with trehalose (Figure 2D), WT-mice treated with metformin (Figure 2E) and APOE-mice treated with metformin (Figure 2F) are presented. There were no significant changes in photoreceptor inner segment/outer segment length (Figure 2G) or the outer nuclear layer (Figure 2H), suggesting loss of photoreceptors does not underlie the changes in photoreceptor function observed in the APOE-mice (Two way ANOVA for genotype and treatment, p > 0.05). Additionally, there was no loss of any retinal layer (data not shown) and total retinal thickness was unchanged between WT- and APOE-mice and drug treated animals (Figure 2I; Two way ANOVA for genotype and treatment, p > 0.05), suggesting that long term treatment with metformin and trehalose does not cause retinal toxicity.

**Table 1.** Optical coherence tomography (OCT) analysis of retinal layer thickness.

Retinal layer	Genotype	Control (µm)	Trehalose (µm)	Metformin (µm)	Statistic (p-value) (genotype/drug)
GCL+IPL	WT	70.7 ± 1.4	71.3 ± 1.4	71.5 ± 0.8	gen, p = 0.065 drug, p = 0.308
	APOE	71.8 ± 1.8	73.1 ± 1.2	73.4 ± 1.3	
INL	WT	28.1 ± 0.5	25.3 ± 0.5	26.1 ± 1.0	gen, p = 0.816 drug, p = 0.060
	APOE	27.2 ± 0.9	25.7 ± 1.2	27.0 ± 1.2	
ONL	WT	76.2 ± 1.1	76.0 ± 0.9	77.0 ± 1.3	gen, p = 0.837 drug, p = 0.309
	APOE	80.6 ± 6.8	73.3 ± 2.0	76.6 ± 2.0	
IS/OS/RPE	WT	38.9 ± 1.3	39.0 ± 1.6	38.5 ± 1.4	gen, p = 0.206 drug, p = 0.200
	APOE	35.8 ± 1.3	39.8 ± 10	38.5 ± 1.4	
Total	WT	213.7 ± 2.3	211.6 ± 2.8	213.2 ± 1.7	gen, p = 0.772 drug, p = 0.470
	APOE	208.7 ± 2.9	212.9 ± 2.4	215.1 ± 3.2	

Retinal layer acronyms: GCL, ganglion cell layer; IPL, inner plexiform layer; INL, inner nuclear layer; ONL, outer nuclear layer; IS, inner segments; OS, outer segments; RPE, retinal pigment epithelium; Total, total retinal thickness

**Figure 3.**
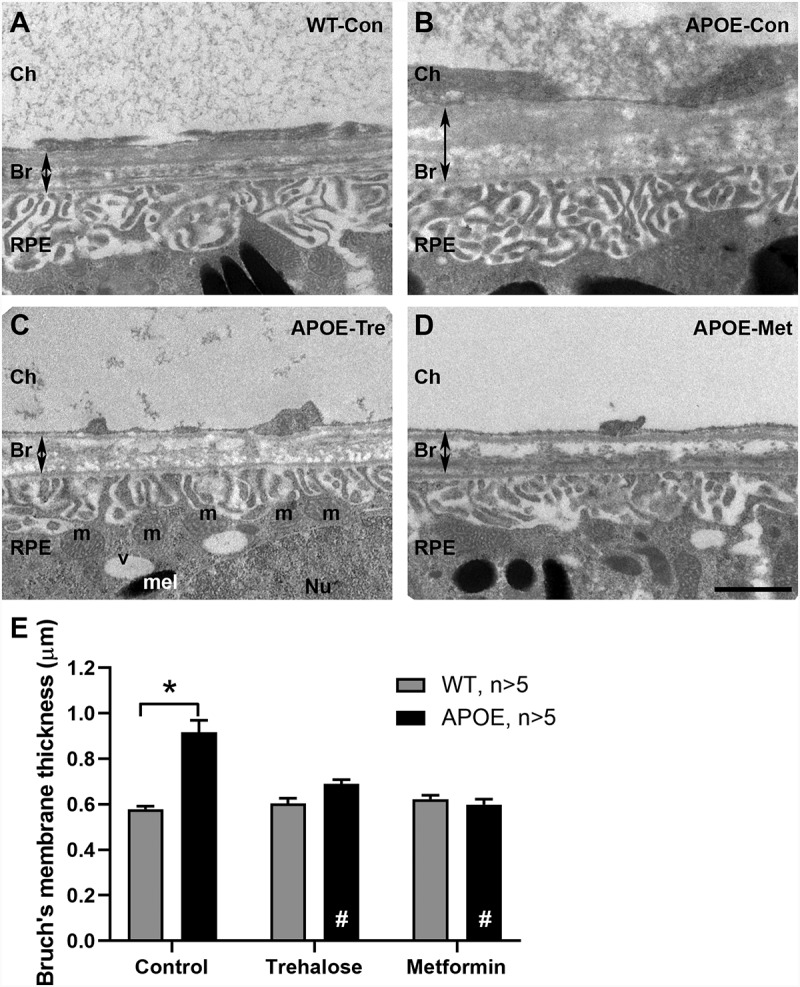
APOE-mice show thickening of Bruch’s membrane at 13 months that is ameliorated by treatment with trehalose or metformin. Bruch’s membrane thickness was investigated using transmission electron microscopy. (A-D) Representative images of Bruch’s membrane are presented for (A) WT-control, (B) APOE-control, and APOE-mice treated with (C) trehalose and (D) metformin. (E) Bruch’s membrane thickness was assessed using segmentation analysis and was found to be significantly thicker in 13-month-old control APOE-mice (black bars) than in age matched WT-mice (gray bars). Treatment of APOE-mice with either trehalose or metformin for 8 months resulted in Bruch’s membrane being similar in thickness to WT-control mice. For all groups n = >5; Two way ANOVA with post-hoc significance of p < 0.05 shown for genotype (*) and treatment (#). Scale: 1 µm. Ch, Choroid; Br, Bruch’s membrane; RPE, retinal pigment epithelium; m, mitochondria; v, vacuole; Nu, nucleus; mel, melanosomes.

Bruch’s membrane, which is a semi-permeable layered collagen membrane involved in nutrient and waste transfer between the choroidal blood supply and the RPE/photoreceptors, was investigated using transmission electron microscopy (Figure 3). This membrane has been found to thicken in early AMD in humans [4–6], contributing to photoreceptor dysfunction in this disease. Representative images of Bruch’s membrane (Br) are presented for WT-control (Figure 3A), APOE-control (Figure 3B) and APOE-mice treated with trehalose (Figure 3C) and metformin (Figure 3D). In 13 month old control APOE-mice, Bruch’s membrane was found to be significantly thicker than in age matched WT-mice (Figure 3E; Two way ANOVA with significance of p < 0.05 for genotype indicated by *). This phenotype is consistent with previous reports of APOE-mice [22] and other mouse models of early AMD [25]. Treatment of APOE-mice with either trehalose or metformin for 8 months abrogated the thickness of Bruch’s membrane observed in APOE-control mice (Figure 3E; Two way ANOVA with significance of p < 0.05 for treatment indicated by #), such that Bruch’s membrane thickness was similar to WT-Control and drug treated WT-mice.

### Autophagy-Lysosomal pathways are impaired in the RPE and retina of APOE-mice and treatment with trehalose or metformin ameliorate aspects of this deficit

**Figure 4.**
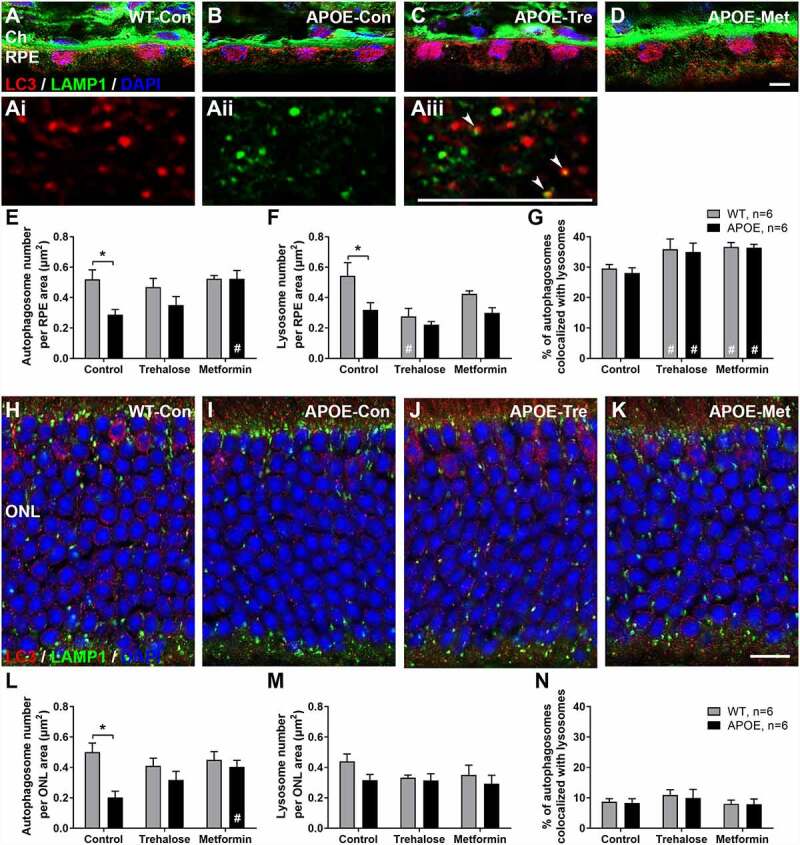
Autophagy-Lysosomal pathways are impaired in the RPE and retina of APOE-mice and treatment with trehalose and metformin ameliorate aspects of this deficit. Autophagy–lysosomal pathways in RPE and photoreceptors were investigated using transverse sections labeled for autophagosomes with an LC3 antibody (red) and lysosomes with a LAMP1 antibody (green). (A-D) Representative images of RPE from (A) WT-control mice, with magnified view of (Ai) LC3-puncta, (Aii) LAMP1-puncta and (Aiii) colocalization (white arrowheads); (B) APOE-control mice; and APOE mice treated with (C) trehalose and (D) metformin are shown. (E) The number of LC3-puncta in the RPE was reduced in APOE-control mice relative to WT-control mice. This loss of LC3-puncta was not apparent in APOE-mice treated with trehalose or metformin relative to their treated WT counterparts. (F) LAMP1-puncta were also reduced in the RPE of APOE-control relative to WT-control mice and this was not altered by treatment with trehalose or metformin. (G) Both trehalose and metformin were found to enhance the number of colocalized LC3- and LAMP1-puncta, suggesting active autophagy was increased in the RPE of both WT and APOE mice relative to their genetic controls. (H-K) Representative images of the photoreceptor nuclei layer in the retina from (H) WT-control mice, (I) APOE-control mice, and APOE mice treated with (J) trehalose and (K) metformin are shown. (L) The number of LC3-puncta in the photoreceptors were reduced in APOE-control mice relative to WT-control mice but not in APOE-mice treated with trehalose or metformin. (M) LAMP1-puncta number and (N) colocalized LC3- and LAMP1-puncta were not altered by either genotype or treatment. For all groups n = >6; Two way ANOVA with post-hoc significance of p < 0.05 shown for genotype (*) and treatment (#). A-D, scale: 5 µm; Ai-Aiii, scale: 5 µm; H-K, scale: 10 µm; RPE, retinal pigment epithelium; ONL, outer nuclear layer.

Disturbances in autophagy-lysosomal degradation within the RPE and also retina have been implicated to play a role in the formation of intracellular waste and subsequent photoreceptor death in AMD and some mouse models [8,9]. To determine whether changes in the autophagy–lysosomal pathways in RPE, photoreceptors, or both contribute to photoreceptor dysfunction in APOE-mice, transverse retinal sections were labeled for autophagosomes with an LC3 antibody (red) and also for lysosomes with a LAMP1 antibody (green, Figure 4). Representative images of RPE from WT-control mice (Figure 4A, with magnified view of LC3-puncta (Ai), LAMP1-puncta (Aii) and colocalization (Aiii)), APOE-control mice (Figure 4B), and APOE mice treated with trehalose (Figure 4C) and metformin (Figure 4D) are shown. In the RPE, punctate LC3 and LAMP1 labeling was apparent throughout the cytoplasm. Additionally, strong LC3 labeling around the RPE nuclei was observed, consistent with previous studies showing a role for nuclear LC3 in some tissues [26]. Both LC3 and LAMP1 labeling were heavily expressed adjacent to Bruch’s membrane, where mitochondria are densely apparent in the RPE (Figure 3C), consistent with a role in mitophagy [27]. LAMP1 labeling was also apparent in the choroid and partly represents nonspecific blood vessel labeling from the secondary antibody, which labels mouse IgG in the serum adhered to the vascular wall. Quantification of the number of LC3-puncta in the RPE was completed by selecting regions of interest, the cytoplasm excluding the nuclei. This revealed a reduction in autophagosome number in the RPE of APOE-control mice relative to WT-control mice (Figure 4E; Two way ANOVA with significance of p < 0.05 for genotype indicated by *). This loss of LC3-puncta was not apparent in APOE-mice treated with trehalose or metformin relative to their treated WT counterparts (Figure 4E; Two way ANOVA, p > 0.05 for trehalose and metformin WT- vs APOE-mice). Additionally metformin treatment significantly enhanced the number of autophagosomes in the RPE relative to untreated APOE-control mice (Two way ANOVA with significance of p < 0.05 for treatment indicated by #), suggesting a rescue of the phenotype. Lysosome numbers were quantified by counting LAMP1-puncta and these were also reduced in the RPE of APOE-control relative to WT-control mice (Figure 4F; Two way ANOVA with significance of p < 0.05 for genotype indicated by *). This was an effect that was not improved by treatment with trehalose or metformin and instead lysosome number was actually reduced by trehalose treatment in WT-mice relative to WT controls. Importantly, both trehalose and metformin were found to enhance the number of colocalized autophagosomes and lysosomes, suggesting active autophagy was increased in the RPE of both WT and APOE mice relative to their genetic controls (Figure 4G; Two way ANOVA with significance of p < 0.05 for treatment indicated by #).

In the retina, autophagosomes, lysosomes and autolysosomes were also investigated histologically and representative images from WT-control mice (Figure 4H), APOE-control mice (Figure 4I), and APOE mice treated with trehalose (Figure 4J) and metformin (Figure 4K) are shown. As was seen in the RPE, the number of LC3-puncta in the photoreceptors were reduced in APOE-control mice relative to WT-control mice (Figure 4L; Two way ANOVA with significance of p < 0.05 for genotype indicated by *). This reduction in autophagosome number was not apparent in APOE-mice treated with trehalose or metformin and metformin treatment significantly enhanced the number of autophagosomes in the retina relative to APOE-control mice (Figure 4L; Two way ANOVA with significance of p < 0.05 for treatment indicated by #). In contrast to the RPE, lysosome number indicated by LAMP1-puncta (Figure 4M) and autolysosome number indicated by colocalized LC3- and LAMP1-puncta (Figure 4N) were not altered by either genotype or treatment.

### Protein array analysis of autophagy pathways in the RPE and retina reveal potential mechanisms by which trehalose and metformin enhance metabolism and autophagy in APOE-treated mice

**Figure 5.**
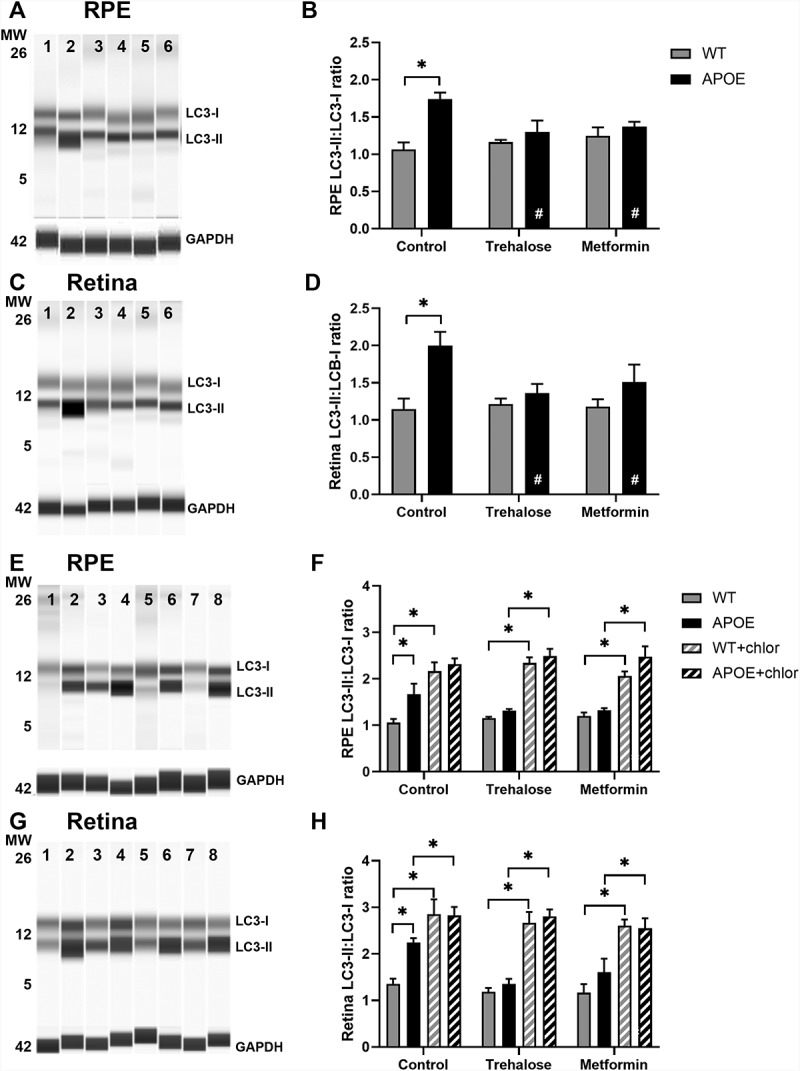
Analysis of LC3 expression shows slowing of autophagy in the RPE and retina of APOE-mice, which is enhanced by treatment with trehalose and metformin. LC3 expression was assessed using simple western and the ratio of LC3-II:LC3-I used as a measure of autophagy. At 5 months of age, WT- and APOE-mice were treated with metformin (0.4 g/kg/day) or trehalose (3 g/kg/day) provided in the drinking water, while sham controls received standard drinking water. At 13 months of age, (A-B) RPE and (C-D) retina samples were analyzed for LC3B. GAPDH was used as a protein loading control. Representative lane results for (A) RPE and (C) retina are presented: MW, molecular weight marker; 1, WT-control; 2, APOE-control; 3, WT-trehalose; 4, APOE-trehalose; 5, WT-metformin; and 6, APOE-metformin. In both (B) RPE and (D) retina, the LC3-II:LC3-I ratio was higher in APOE-mice suggesting a slowing of autophagy. In APOE animals treated with trehalose or metformin the ratio of LC3-II:LC3-I was restored to WT levels. (E-H) To assess autophagy flux, experiments to block autophagy-lysosomal degradation were completed using chloroquine. At 5 months of age, WT- and APOE-mice were treated for 2 weeks with metformin or trehalose and then retinae and RPE/choroid/sclera complex were incubated in culture media with or without 50 µM chloroquine for 24 h. Example results for E) RPE and (G) retina from WT and APOE mice are presented: Lanes 1, WT-control; 2, WT-control + chloroquine, 3, APOE-control; 4, APOE-control + chloroquine; 5, APOE-trehalose; 6, APOE-trehalose + chloroquine; 7, APOE-metformin; 8, APOE-metformin + chloroquine. The LC3-II:LC3-I ratio in (F) RPE and (H) retinal samples increased significantly in all samples treated with chloroquine to block autophagy-lysosomal function. For all groups, n = 5; Two way ANOVA with post-hoc significance of p < 0.05 (*).

**Figure 6.**
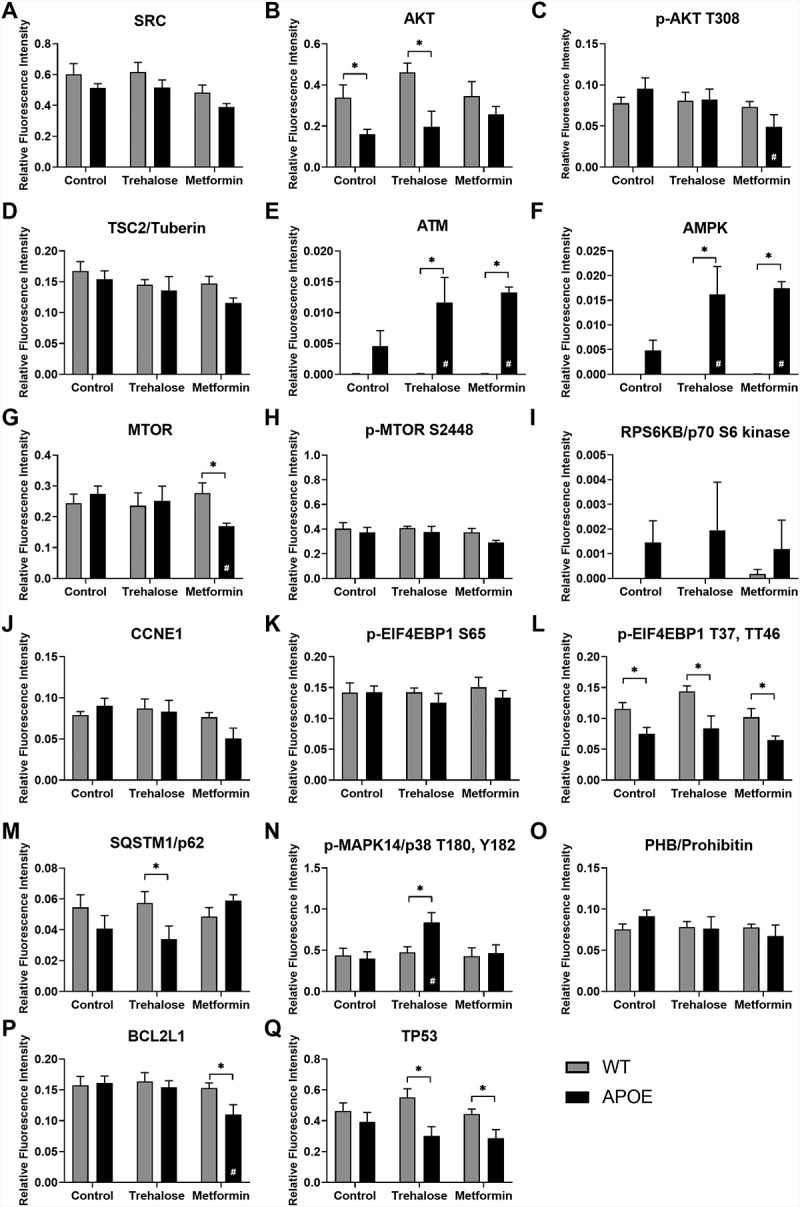
Protein array analysis of autophagy pathways in the RPE. Reverse phase protein array analysis was used to quantify protein expression changes in the autophagy pathways in the RPE from WT- (gray bars) and APOE-mice (black bars) treated with trehalose and metformin. Results are shown for (A) SRC, (B) AKT, (C) AKT phosphorylated at T306, (D) TSC2/tuberin, (E) ATM, (F) AMPK, (G) MTOR, (H) MTOR phosphorylated at S2448, (I) RPS6KB/p70 S6 kinase, (J) CCNE1, (K-L) EIF4EBP1 phophorylated at (K) S65 and (L) T37, T46, (M) SQSTM1/p62, (N) MAPK14/p38 phosphorylated at T180, (O) PHB, (P) BCL2L1, and (Q) TP53. For all groups, n = 5; Two way ANOVA with post-hoc significance of p < 0.05 shown for genotype (*) and treatment (#).

**Figure 7.**
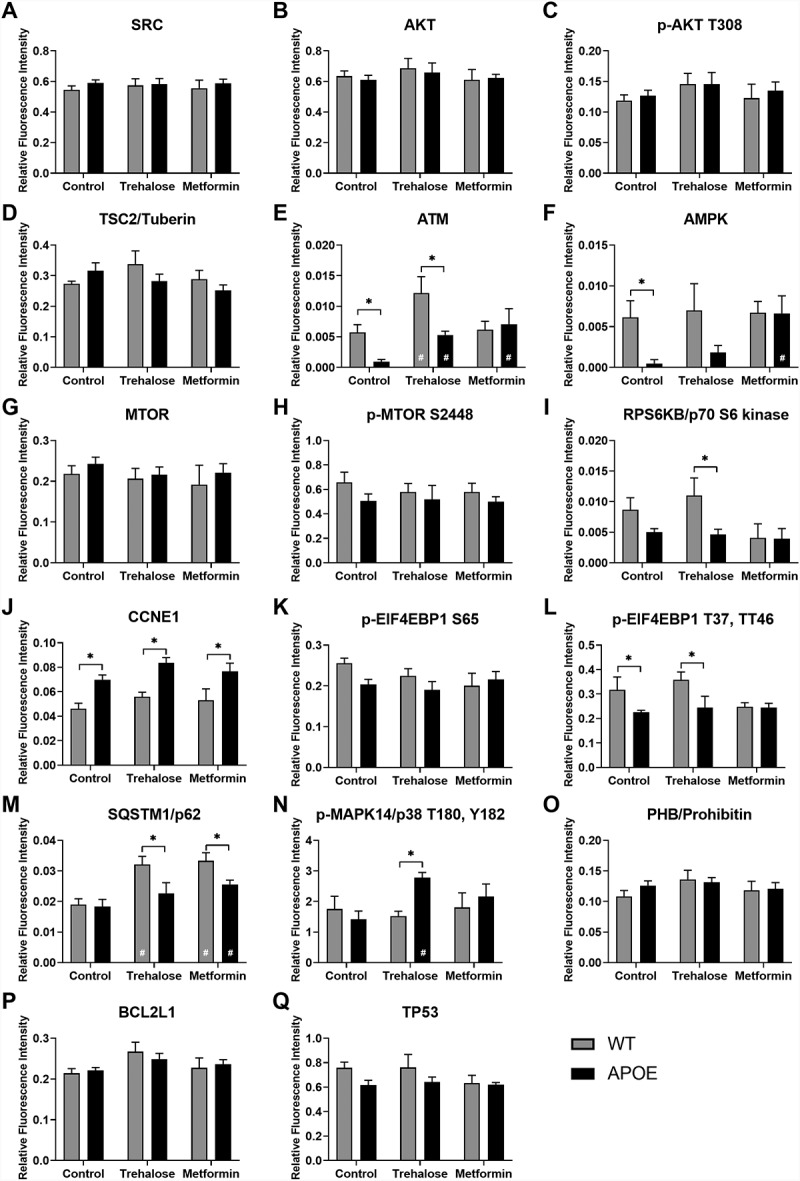
Protein array analysis of autophagy pathways in the whole retina. Reverse phase protein array analysis was used to quantify protein expression changes in the autophagy pathways in the retina from WT- (gray bars) and APOE-mice (black bars) treated with trehalose and metformin. Results are shown for (A) SRC, (B) AKT, (C) AKT phosphorylated at T306, (D) Tuberin (TSC2 complex), (E) ATM, (F) AMPK, (G) MTOR, (H) MTOR phosphorylated at S2448, (I) RPS6KB/p70 S6 kinase, (J) CCNE1, (K-L) EIF4EBP1 phophorylated at (K) S65 and (L) T37, T46, (M) SQSTM1/p62, (N) MAPK14/p38 phosphorylated at T180, (O) PHB, (P) BCL2L1, and (Q) TP53. For all groups, n = 5; Two way ANOVA with post-hoc significance of p < 0.05 shown for genotype (*) and treatment (#).

**Figure 8.**
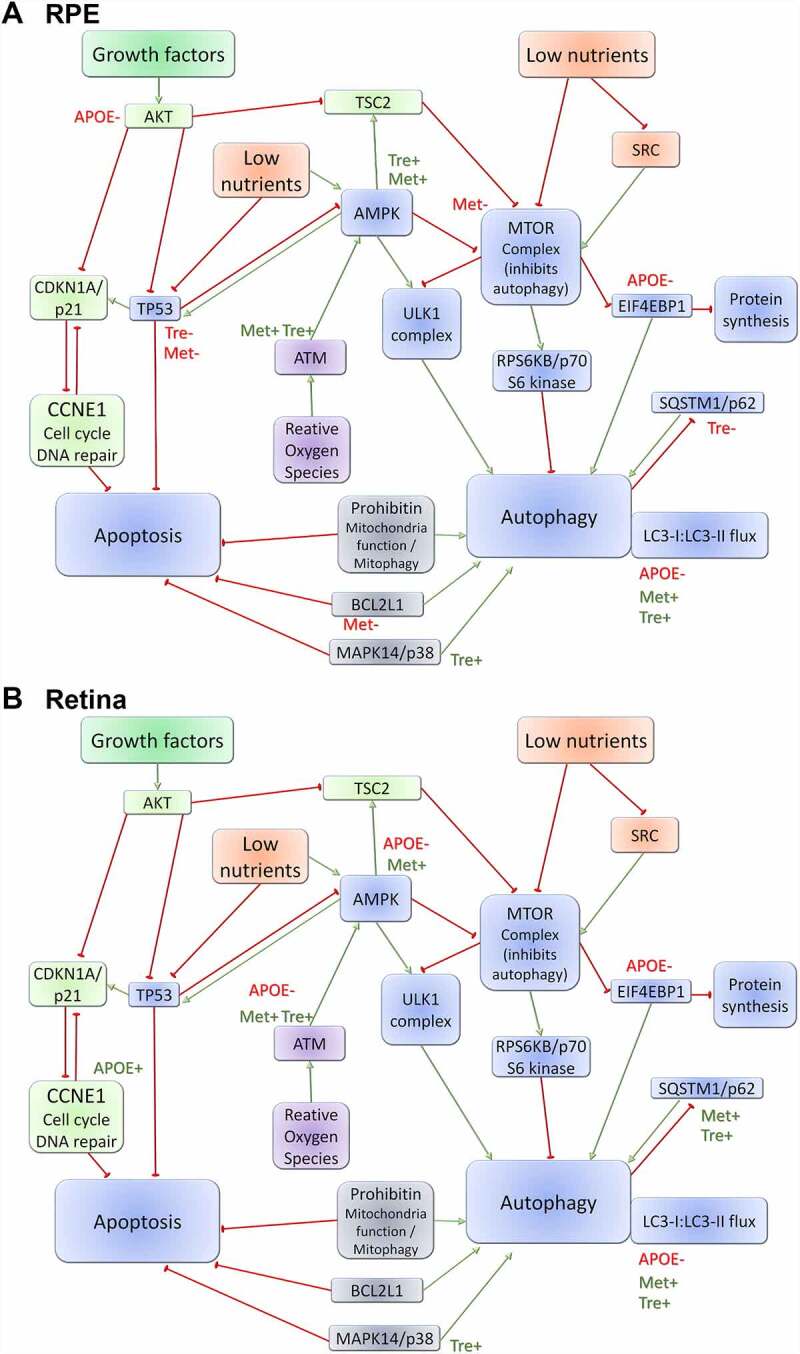
Schematic of the autophagy protein pathways investigated. A combination of reverse phase protein array (RPPA) and Western protein analysis were used to quantify protein expression changes in the autophagy pathways in the (A) RPE and (B) retina from WT- and APOE-mice treated with trehalose and metformin and the results from this analysis are summarized in diagram form. Growth factor pathways are indicated in green; low nutrient pathways are indicated in Orange; reactive oxygen species pathways are indicated in purple; and intersecting pathways are indicated in blue. Green arrows indicate pathway upregulation; red bubble arrows indicate pathway down-regulation; green text (+) indicates upregulated in genotype/treatment group; and red text (-) indicates down-regulated in genotype/treatment group.

A combination of reverse phase protein array and simple western protein analysis were used to quantify protein expression changes in the autophagy pathways in the RPE and retina from WT- and APOE-mice treated with trehalose or metformin. For LC3-II:LC3-I expression, a central protein in the autophagy pathway involved in substrate selection and autophagosome biogenesis, simple western results are presented in Figure 5 for aged RPE (Figure 5A,B) and aged retina (Figure 5C,D) and for autophagy flux experiments (Figure 5E-H). The remainder of proteins were assessed using reverse phase protein array and results are shown for the RPE (Figure 6) and retina (Figure 7), for (A) SRC, (B) AKT, (C) AKT phosphorylated at T306, (D) TSC2/tuberin, (E) ATM, (F) AMPK, (G) MTOR, (H) MTOR phosphorylated at S2448, (I) RPS6KB/p70 S6 kinase, (J) CCNE1, (K-L) EIF4EBP1 phophorylated at (K) S65 and (L) T37, T46, (M) SQSTM1/p62, (N) MAPK14/p38 phosphorylated at T180, (O) PHB/prohibitin, (P) BCL2L1/Bcl-xL, and (Q) TP53. The results from this analysis are summarized in diagram form in Figure 8.

In the RPE, APOE-mice mice showed an increased ratio of LC3-II:LC3-I (Figure 5A,B) and reduced expression of AKT (Figure 6B), and EIF4EBP1 phophorylated at T37, T46 (Figure 6L), suggesting slowed autophagy processes in APOE-control mice relative to WT-mice (Two way ANOVA with significance of p < 0.05 for genotype indicated by *). While treatment of APOE-mice with trehalose did not improve expression levels of AKT (Figure 6B) or EIF4EBP1 phophorylated at T37, T46 (Figure 6L) relative to WT-mice (Two way ANOVA with significance of p < 0.05 for genotype indicated by *), the ratio of LC3-II:LC3-I was reduced to WT-levels and was significantly reduced relative to APOE-control mice (Figure 5A,B; Two way ANOVA with significance of p < 0.05 for treatment indicated by #). The mechanisms by which trehalose enhances LC3-II turnover may involve increasing expression of ATM (Figure 6E) and subsequently AMPK (Figure 6F), however, this pathway was independent of MTOR complex 1 (MTORC1) expression changes (Figure 6G,H). Additional separate pathways involving increasing MAPK14/p38 expression (Figure 6N) and reducing TP53/TRP53/p53 (the mouse protein is TRP53, but TP53 is uesd for simplicity; Figure 6Q) expression may also contribute to enhanced LC3-II turnover in RPE of APOE-mice treated with trehalose (Two way ANOVA with significance of p < 0.05 for genotype indicated by * and treatment by #). Similar to trehalose, treatment of APOE-mice with metformin did not improve expression levels of EIF4EBP1 phophorylated at T37, T46 (Figure 6L) relative to WT-mice (Two way ANOVA with significance of p < 0.05 for genotype indicated by *), however, AKT levels were enhanced (Figure 6B) and the ratio of LC3-II:LC3-I was reduced to WT-levels (Figure 5A,B; Two way ANOVA with significance of p < 0.05 for genotype indicated by * and for treatment indicated by #). Metformin may enhance LC3-II turnover by increasing expression of ATM (Figure 6E) and subsequently AMPK (Figure 6F), causing subsequent inhibition of MTOR complex expression (Figure 6G,H). Like trehalose, metformin may also enhance autophagy processes by reducing TP53 levels (Figure 6Q). Autophagy flux experiments were completed using the autophagy-lysosomal blocker chloroquine. Chloroquine treatment induced a significant increase in the LC3-II:LC3-I ratio in all groups, suggesting autophagy-lysosomal processes could be futher blocked (Figure 5E,F). This indicates autophagy degradation processes are still occurring in the APOE RPE, even though they are slowed relative to WT, and that trehalose and metformin are enhancing LC3-II turnover in APOE tissues.

In the retina, APOE-mice mice showed multiple protein expression changes consistent with a reduction in autophagy, including reduced expression of ATM (Figure 7E), and subsequent reduction of AMPK (Figure 7F) but without affecting the MTOR complex protein expression levels relative to WT (Figure 7G,H; Two way ANOVA with significance of p < 0.05 for genotype indicated by *). Like the RPE, there was reduced EIF4EBP1 phophorylated at T37, T46 (Figure 7L) but also an increase in CCNE1 (Figure 7J). Ultimately, an increased ratio of LC3-II:LC3-I was also observed suggesting slowed autophagy processes in the retina of APOE-control mice relative to WT-mice (Figure 5C,D; Two way ANOVA with significance of p < 0.05 for genotype indicated by *). Trehalose treatment of APOE mice did not alter the expression changes in EIF4EBP1 phophorylated at T37, T46 (Figure 7L) or CCNE1 (Figure 7J) relative to WT-mice (Two way ANOVA with significance of p < 0.05 for genotype indicated by *). However the ratio of LC3-II:LC3-I was reduced to WT-levels and was significantly reduced relative to APOE-control mice (Figure 5C,D; Two way ANOVA with significance of p < 0.05 for treatment indicated by #). These changes in LC3 turnover may have been impacted by trehalose via a few different pathways. Trehalose mildly but significantly enhanced ATM expression in APOE-mice relative to APOE-controls (Figure 7E), significantly increased expression of phosphorylated MAPK14/p38 (Figure 7N) and significantly reduced RPS6KB/p70 S6 kinase expression relative to treated WT-mice (Figure 7I). Like trehalose, meformin treatment of APOE mice showed some benefit in modulating expression of proteins driving autophagy processes. While, metformin did not alter the expression changes in CCNE1 relative to WT-mice (Figure 7J; Two way ANOVA with significance of p < 0.05 for genotype indicated by *), metformin treatment of APOE-mice did increase expression of ATM (Figure 7E) and subsequently AMPK (Figure 7F) relative to untreated APOE-control mice (Two way ANOVA with significance of p < 0.05 for treatment indicated by #). These changes occurred independently of MTOR pathway changes (Figure 7G,H), but are likely to have contributed to the reduction in LC3-II:LC3-I ratio observed in metformin treated APOE-mice relative to APOE-controls (Figure 5C,D; Two way ANOVA with significance of p < 0.05 for treatment indicated by #). Autophagy flux experiments using chloroquine showed a significant increase in the LC3-II:LC3-I ratio in all groups after lysosomal blockade (Figure 5G,H), indicating autophagy degradation processes are still occurring in the APOE retina, and that trehalose and metformin enhance LC3-II turnover in APOE retina. Interestingly both trehalose and metformin generally increased SQSTM1/p62 expression in the retina of both WT and APOE-mice relative to untreated controls (Figure 7M; Two way ANOVA with significance of p < 0.05 for genotype indicated by * and treatment indicated by #), suggesting that while autophagy was generally enhanced this may have come at the expense of processing of ubiquinated proteins tagged with SQSTM1/p62 for degradation, so that autophagy pathways to enhance metabolic function were prioritized instead.

## Discussion

There is increasing evidence suggesting a role for autophagic impairment in a wide range of age-related neurodegenerative diseases including AMD. In this study we investigated treatments targeting autophagy as potential therapies for slowing development of an early AMD phenotype in mice lacking APOE. Thirteen month old APOE mice had thickened Bruch’s membrane and photoreceptor dysfunction consistent with an early AMD phenotype. We show for the first time that photoreceptor dysfunction occurs in this mouse model at this age and that this is associated with changes in autophagy pathways in both the retina and RPE. Treatment with either metformin or trehalose for 8 months ameliorated the early AMD phenotype in APOE mice and restored autophagy LC3-II:LC3-I ratio to WT levels with an increase in LC3-puncta apparent in the photoreceptors and RPE of treated APOE mice. Both treatments modulate the ATM-AMPK pathway that is usually upregulated in times of metabolic insufficiency to enhance autophagy for the purposes of cell survival. These data provide support for the use of drugs targeting this pathway in the treatment of AMD.

While mice lack a macular region, they can be used to model key mechanistic pathways where global changes are apparent that are consistent with those observed in humans with early AMD. In line with this, APOE mutant mice have been shown to be a useful model of early AMD [20,21] and for assessing AMD autophagy changes in the RPE when fed a high-fat diet [8]. Our previous work has shown that they may be a useful model of early AMD without requiring a high fat diet, showing thickened Bruch’s membrane and accumulation of lipid deposits in the basal RPE and Bruch’s membrane by 13 months [18,22]. Additionally, in the current study, APOE mice were found to have deficits in photoreceptor function at 13 months, which occurred without photoreceptor loss. This is similar to findings in humans who show loss of scotopic sensitivity and dark adaptation with normal aging [28], but have a significantly greater loss of function in the early stages of AMD [29–31]. While dark adaptation changes are not as apparent in mice with age, loss of photoreceptor function, as measured by reduction in the ERG a-wave, does occur [32]. Commonly a loss of rod photoreceptor function occurs due to a reduction in photoreceptor numbers or in inner/outer segment length [33], however, at a histological level the photoreceptors remained, suggesting changes at the subcellular level in the retina and RPE may contribute to the functional deficits in APOE mice. A similar early AMD phenotype has been observed in aged *p2rx7/p2x7* null mice, where an increase in Bruch’s membrane thickness and loss of retinal function occurred without loss of photoreceptors [25]. Reductions in photoreceptor function may occur due to thickening of Bruch’s membrane, limiting nutrient and oxygen transfer from the ocular blood supply to the RPE and neural retina. Additionally, our data also suggest changes in metabolism and autophagy within the retinal neurons and RPE may underlie dysfunction causing functional deficits prior to cell loss.

In both the photoreceptors and RPE of APOE mice, histological and protein expression analysis indicated a slowing of autophagy relative to WT controls. Immunohistochemical analysis showed a significant reduction in autophagosome numbers (LC3-positive puncta) in the RPE and photoreceptors of APOE mice relative to WT. In the RPE also there was a significant reduction in LAMP1-positive puncta suggestive of a reduction in the number of lysosomes. This correlated with an increased ratio of LC3-II:LC3-I in the APOE RPE and retina, suggesting a slowing of autophagy. However, there was no change in autophagosome fusion with lysosomes, as LC3 colocalization with LAMP1-puncta was not altered in either the RPE or retina of APOE mice, suggesting basal levels of autophagic-lysosomal digestion were occurring but processes prior to this step were slowed. A similar phenomenon of slowing autophagy has been observed in the retina of CLN6 mutant mice, which have a lysosomal storage disorder and undergo early retinal degeneration [34]. A decrease in LC3-puncta in photoreceptors was found to correlate with an increased LC3-II:LC3-I ratio in the retina of this mouse model [34]. Further molecular pathway analysis at the protein level in the retina of APOE mice mice showed expression changes consistent with the observed reduction in autophagy, including reduced expression of ATM and AMPK. This pathway is involved in energy sensing and response to reactive oxygen species [35]. Reductions in the AMPK pathways have been found to occur in the retina with age and loss of these pathways models aging effects in young animals [36]. Additionally, EIF4EBP1 phophorylated at T37 and T46 was down-regulated in both the retina and RPE of 13 month old APOE mice. This protein is downstream of MTOR and acts as an enhancer of autophagy at the expense of protein synthesis [37–39]. Down regulation of phosphorylated EIF4EBP1 indicates another step in the pathway by which autophagy may be slowed in the retina and RPE of APOE mice.

Trehalose, a non-reducing disaccharide, and metformin, the type II diabetes drug, have both been found to enhance autophagy and were investigated for their potential to modulate visual dysfunction in aging WT and APOE mice. WT and APOE mice were treated from 5 months of age and ocular morphology, function and autophagy pathways investigated at 13 months. Neither drug had an effect on retinal morphology in either WT or APOE mice suggesting these drugs were not overtly toxic to the eye when administered at the present doses over a long time. Indeed, metformin was found to induce a subtle but significant increase in rod photoreceptor and cone pathway function in treated WT-mice relative to untreated WT-controls at 13 months. As retinal function in the WT (C57BL/6 J) mouse has been found to decline with age [32], it is tempting to suggest that long term treatment with metformin may have a protective effect against age-induced visual decline. Metformin has been shown to extend lifespan in mice [15], suggesting this is a possibility. Additionally, metformin and also trehalose had a protective effect on retinal function in APOE mice. While retinal function was reduced in APOE mice, this was not apparent in APOE mice treated with trehalose or metformin, suggesting these treatments may be used to slow retinal dysfunction in this model of early AMD. This is consistent with previous studies that show metformin can rescue retinal dysfunction in a light damage model of retinal degeneration [9].

The autophagy and metabolic pathways by which metformin and trehalose modulated ocular morphology and retinal function were investigated by a combination of histological and protein pathway analysis. Histological analysis showed that while loss of LC3-puncta was apparent in both the photoreceptors and RPE of APOE mice compared with WT mice, there was no reduction in LC3-positive puncta in these tissues of treated APOE mice relative to their WT controls. This correlated with a reduction in the LC3-II:LC3-I ratio in treated APOE mice, suggesting that autophagy processes were enhanced in both the retina and RPE of APOE mice treated with metformin or trehalose. Additionally, in the RPE of treated WT and APOE mice there was an increase in LC3-puncta fusion with LAMP1-puncta, suggesting autophagic-lysosomal digestion may have been enhanced above basal levels in mice treated with metformin or trehalose. A decrease in LC3-puncta fusion with LAMP1-puncta has been noted in mice with genetic dysfunction of lysosmal storage [34], thus an increase in this process in the RPE of both treated WT and APOE mice, suggests that these drugs may aid the waste processing functions of the aging RPE.

Subsequent detailed protein pathway analysis using RPPA indicated that both metformin and trehalose modulate the ATM-AMPK pathway to modify autophagy in the retina and RPE of APOE mice (Figure 8). The ATM-AMPK pathway is stimulated to enhance autophagy in response to energy deficiencies and oxidative stress, promoting cell survival [40]. APOE mice heterozygous for ATM display accelerated metabolic syndrome and atherosclerosis, indicating that genetic suppression of the ATM-AMPK pathway contributes to the development of dysfunction [41]. In the retina, the PRKAA2/AMPKα2 protein has been specifically implicated in the pathways that modulates dysfunction. Metformin has been shown to slow retinal degeneration in a range of models via stimulation of PRKAA2/AMPKα2 to enhance metabolic function in both the retina and RPE [9]. Our findings are consistent with this previous work and highlight the potential of ATM-AMPK pathway modulators in slowing retinal dysfunction prior to degeneration. Additionally, both metformin and trehalose were found to down-regulate TP53 expression in the RPE. TP53 has been shown play a delicate role in modulating autophagy versus apoptosis [42]. Increases in TP53 have been shown to inhibit AMPK and promote apoptosis [42], thus it is possible that metformin and trehalose may also promote AMPK via inhibition of basal TP53 expression in the RPE.

Like TP53, MAPK14/p38 has been shown to play a role in regulating the balance of autophagy versus apoptosis, with up-regulation shown to induce survival promoting autophagy [43]. Trehalose was found to up-regulate phosphorylated MAPK14/p38 in both the retina and RPE. Thus treatment with trehalose may have additional benefits over metformin by promoting autophagy via an additional AMPK independent pathway.

Changes in SQSTM1/p62 were also encountered with both trehalose and metformin increasing SQSTM1/p62 expression in the retina in WT and APOE -mice relative to untreated controls, while trehalose reduced SQSTM1/p62 expression in the RPE of APOE mice. SQSTM1/p62 is involved with tagging ubiquinated protein aggregates for degradation by autophagy and this protein accumulates when autophagy-lysosomal processes are blocked. SQSTM1/p62 has been shown to accumulate in the RPE and retina of mice with deficiencies in lysosomal storage and in WT retina following treatment with the autophagy-lysosomal blocker, chloroquine [34]. The accumulation of SQSTM1/p62 in the retina of animals treated with trehalose and metformin suggests that while autophagy was generally enhanced as determined from the LC3-II:LC3-I ratio, this may have come at the expense of degradation of SQSTM1/p62 tagged protein aggregates. SQSTM1/p62 has been shown to be a poor indicator of autophagy under long term starvation conditions [44,45]. As both trehalose and metformin modulate energy deficiency autophagy pathways in a manner similar to starvation, it is possible that long term treatment with these drugs enhance metabolic autophagy separately from the characteristic SQSTM1/p62 protein aggregate autophagy response.

Overall, the findings of this study highlight the potential of drugs targeting autophagy as treatments for slowing the development of AMD. In line with this, metformin has recently been shown to reduce the risk of AMD development in humans [17]. In elderly patients, greater than 55 years of age from Florida, metformin treatment was associated with an odds ratio of 0.58 of developing AMD independently of diabetes which also seemed to be protective against development of AMD (0.32 odds ratio) [17]. Future work investigating the benefits of therapies targeting metabolism and autophagy in mouse models and clinical trials may elucidate a treatment for slowing the progression of early AMD to vision threatening disease.

## Methods

***Animals:*** Homozygous B6.129P2- APOE^*tm*1*Unc*^J/Arc on a C57BL/6 J-background, which lack APOE (APOE-mice), and C57BL/6 J- (wild-type, WT) control mice were obtained from the Animal Resources Center (Western Australia; catalog numbers: APOE and B6) at 6 to 8 weeks of age. Animals were housed at the Biomedical Sciences Animal Facility at the University of Melbourne under a 12:12 h light-dark cycle with *ad libitum* access to food and water. At 5 months of age mice were treated with metformin (0.4 g/kg/day; LKT Laboratories Inc., M2076) or trehalose (3 g/kg/day; Sigma-Aldrich/Merck, T9449) provided in the drinking water, while sham controls received standard drinking water. Doses were selected based on previous studies showing effects on autophagy in mice with these treatments [46–48]. Mice were monitored for weight and health bi-weekly and treated until 13 months of age. This produced 6 groups at the end of the experiment with nearly equal numbers of male and female mice: WT-control (n = 21), WT-trehalose (n = 21), WT-metformin (n = 22), APOE-control (n = 21), APOE-trehalose (n = 21), and APOE-metformin (n = 22). All experiments and handling of animals were conducted in compliance with the standards of the Association of Vision Research and Ophthalmology Statement for the Use of Animals in Ophthalmic and Vision Research as well as the institutional guidelines of The University of Melbourne Animal Ethics Committee; Ethics ID 1513553).

***Fundus imaging and Optical Coherence Tomography (OCT****): In vivo* imaging was used to investigate ocular fundus appearance and retinal thickness in WT- and APOE-retinae at 5 and 13 months of age using spectral domain optical coherence tomography (SD-OCT; Phoenix Research Labs, Pleasanton, CA, USA) with guidance by live fundus imaging (Micron III® retinal imaging microscope) [49]. Mice were deeply anaesthetized by an intraperitoneal injection of a mixture of ketamine (67 mg/kg; Provet, KETAI1) and xylazine (13 mg/kg; Troy Laboratories, ilium Xylazil-100) and the corneal reflex was supressed by applying 0.5% proparacaine hydrochloride (Alcon Laboratories, Alcaine ® Solution 0.5%). The pupils were dilated by topical application of 1% atropine sulfate (Alcon Laboratories, Isopto® Atropine 1%) and 2.5% phenylephrine hydrochloride (Bausch &Lomb, Minims ® Phenylephrine hydrochloride). A custom-made holder was used to orientate the mouse so that the lens of the Micron III microscope could be applied close to the cornea. The retinal fundus of each mouse was viewed and images captured using the Micron III software. Retinal structure was evaluated using OCT. Linear scans composed of an average from 50 frames were captured across the center of the eye near the optic nerve. Images (n = >5 eyes) were exported as tagged image files (.Tif) and retinal thickness was quantified using custom segmentation software in ImageJ v1.47 (NIH, Bethesda, MD, USA).

***Electroretinography to assess rod and cone pathway function***: Retinal function of the rod and cone pathway of WT- and APOE-mice were assessed at 13 months of age using a twin-flash electroretinogram (ERG). Animals were dark adapted overnight and the next day, under dim red-light, mice were anaesthetised (ketamine, 67 mg/kg; xylazine, 13 mg/kg). The corneal reflex was anaesthetised (0.5% proparacaine hydrochloride) and pupils dilated (1% atropine sulfate; 2.5% phenylephrine hydrochloride). Full field ERG responses were evoked using a commercial flash unit (Nikon SB900, NSW, Australia) and custom made Ganzfeld. Two 2.1 log cd second/m^2^ full-field flashes were delivered with a 0.8s inter stimulus interval, to elicit responses from the rod and cone pathways (mixed response) and the cone pathway (cone response) alone. The responses were captured using custom made Ag/AgCl electrodes placed on the cornea, amplified (gain x5000; −3 dB at 1 Hz and 1 kHz, ML132 BioAmp; ADInstruments, NSW, Australia) and acquired at a 10 kHz sampling frequency over a 250 millisecond period (ML785 Powerlab/8sp amplifier; ADInstruments). Provision of the stimulus and recording of the ERG was coordinated by Scope software version 3.6.10 (ADInstruments).

For ERG analysis, the rod pathway response was generated by digital subtraction of the cone ERG from the mixed ERG signal and various component waveforms assessed as previously described [25,50]. The rod photoreceptor response (rod a-wave) was isolated and analyzed using a modified PIII model to derive the response amplitude (PIII Rmax in μV) and sensitivity (S in m^2^cd^−1^s^−3^). The rod post-photoreceptoral function (rod b-wave) was fitted using an inverted gamma function to derive the amplitude of the PII response (rod PII Rmax in μV) and the time to peak (Implicit time in ms). To assess the oscillatory potentials (OPs) the fitted rod PII was subtracted from the raw b-wave, and the amplitude and time to peak of OPs 2, 3 and 4 were measured and summed. Cone pathway responses were modeled and waveform components for the post-photoreceptor responses, the cone b-wave (PII) and oscillatory potentials (summed OPs 1, 2 and 3) were assessed. Differences in genotype and age were analyzed in GraphPad prism v8 (GraphPad, CA, USA) for each rod and cone pathway ERG component using a two-way ANOVA for genotype and treatment. Post-hoc comparisons were made using a Tukey’s test and p < 0.05 considered significant.

***Histology and transmission electron microscopy***: To assess the structure of the neural retina, RPE and Bruch’s membrane, histology was undertaken [25,50]. Retinal morphology and layer thickness was assessed in WT- and APOE-mice at 13 months of age (n ≥ 5 each group) using toluidine blue stained resin sections. Mice were deeply anaesthetised, euthanized by cervical dislocation and the posterior eye cups were fixed overnight in 1% paraformaldehyde (Sigma-Aldrich/Merck, 158,127–500 G), 2.5% glutaraldehyde (Sigma-Aldrich/Merck, 820,603.1000), 3% sucrose (ChemSupply, SA030), and 0.01% calcium chloride (ChemSupply, CA033-500 G) in 0.1 M phosphate buffer, pH 7.4 (PB). The eyecups were washed in cacodylate buffer (Sigma-Aldrich/Merck, C0250), incubated in 0.5% OsO_4_ (Sigma-Aldrich/Merck, O5500) for 1 h and dehydrated using increasing concentrations of methanol (75%, 85%, 95%, and 100%) and acetone (100%). Samples were then incubated in a mixture of Epon resin (Procure 812, [ProSciTech, C045]; dodecenyl succinic anhydride, DDSA, [ProSciTech,C044]; DMP-30, [ProSciTech, C053-25]), embedded and polymerized at 60°C overnight. Semi thin sections (1-µm thickness) of the entire retina near the optic nerve were prepared using an ultramicrotome (Reichert-Jung Ultracut S; Reichert, Depew, NY) and collected on glass slides. Samples were stained with 1% toluidine blue and mounted with resin and a glass coverslip. An Axioplan microscope (Carl Zeiss, Göttingen, Germany) was used to view retinal sections (X40 magnification under oil), and images were captured using a digital camera and computer software (SPOT, version 3.5.2; Diagnostic Instruments, Victoria Park, WA, Australia). Segmentation analysis, as was applied to optical coherence tomography images, using a custom segmentation script for ImageJ v1.47, was used to determine the thickness (µm) of the ganglion cell layer (GCL), inner plexiform layer (IPL), inner nuclear layer (INL), outer nuclear layer (ONL), inner and outer photoreceptor segments (IS/OS) and total retina.

For analysis of Bruch’s membrane, transmission electron microscopy was used [25,50]. Ultrathin sections were cut on the ultramicrotome at 70 nm. Section were collected on copper grids (ProSciTech, CGu50), contrasted with uranyl acetate and lead citrate solutions and viewed with a Phillips CM120 electron microscope (Field Electron and Ion Company, Hillsboro, OR). Bruch’s membrane was imaged at X14000 magnification. Bruch’s membrane was classified as consisting of five layers: basement membrane of the RPE, inner collagen, elastin layer, outer collagen and basement membrane of the choroid [51]. Segmentation analysis was used to determine changes in Bruch’s membrane thickness. Differences in genotype and treatment for each retinal layer and Bruch’s membrane were analyzed using a two-way ANOVA including a Tukey multiple comparisons posttest (GraphPad prism, p < 0.05). Data are presented for the central retina, as no effect of eccentricity was observed (data not shown).

***Immunohistochemistry and super resolution confocal microscopy***: For histological analysis of autophagy-lysosomal pathways in the retina and RPE, 13 months old WT- and APOE-mice were sacrificed at 11–12 pm. Posterior eyecups were fixed for 30 min in 4% paraformaldehyde (Sigma-Aldrich/Merck, 158,127–500 G) in PB, washed and cryoprotected in a series of sucrose (ChemSupply, SA030) solutions (10%, 20%, 30% v:v in PB). After 24 h, the eyecups were embedded in Optimal Cutting Temperature medium (O.C.T Tissue-Tek, ProSciTech, IA018) and frozen. Retinal sections were cut transversely at 14 µm on a cryostat (Leica CM1860 UV, Wetzlar, Germany), collected onto poly-L-lysine-coated slides and stored at −30°C until further use. For labeling, slides were defrosted, washed in PB and incubated overnight with either a single or a combination of primary antibodies diluted in antibody buffer (3% v:v normal goat serum [ThermofischerScientific, 31,872]; 1% w:v bovine serum albumin [Sigma-Aldrich/Merck, A2153]; 0.5% v:v Triton X-100 [Sigma-Aldrich/Merck, X100] in PB). To label autophagosomes, a polyclonal rabbit antibody against MAP1LC3B/LC3 generated against a synthetic peptide to the N-terminal portion of the human LC3 protein sequence was used (1:300; Novus Biologicals, NB100-2220). To label lysosomes, a monoclonal rat antibody against LAMP1 generated against NIH/3T3 mouse embryonic fibroblast tissue culture cell membranes was used (1:50; Developmental Studies Hybridoma Bank, 1D4B). Sections were rinsed in PB and incubated for 90 min with the nuclear dye 4’,6-diamidino-2-phenylindole (DAPI at 1:1000; Thermofisher Scientific, D1306) and secondary antibodies as required: goat anti-rat, or goat anti-rabbit conjugated to Alexa Fluor 488 or Alexa Fluor 594, (1:500; Thermofisher Scientific, A-11006 and A-11012). Sections were washed, covered with Dako mounting medium (Agilent Technologies, S3023) and a glass coverslip.

Transverse retinal and RPE images were collected using a super resolution confocal laser scanning microscope in Airy scan mode (LSM 800, Carl Zeiss AG, Oberkochen, Germany), with a X63 oil immersion objective at a resolution of 2048 × 2048 pixels. After acquisition, images were deconvolved using the airyscan processing software (ZEN blue, Carl Zeiss AG). Images were edited using LSM Zeiss software, ImageJ, or Adobe Photoshop CC (Adobe Systems Incorporated, San Jose, CA, USA). For analysis image quality was optimized similarly for WT and APOE-tissues by adjusting the following parameters: laser power, gain, and black levels.

Autophagosomes, labeled with an LC3 antibody; lysosomes, labeled with a LAMP1 antibody; and autolysosomes, colocalization of LC3 and LAMP1, were quantified in tissues from WT- and APOE-mice (n = 6/ genotype/treatment) using previously published techniques [34]. Two super resolution Z-stack images per animal were collected for the RPE and the outer nuclear layer and were assessed using a custom script in FIJI to quantify autophagosome and lysosome number (puncta number/area of region of interest μm^2^), average puncta size (μm^2^), as well as colocalization of autophagosomes and lysosomes in each retinal layer. Confocal images were automatically re-named using a file naming script and the analysis was done by a researcher, who was blind to the experimental group. Regions of interest were created to define the individual retinal layers. Local maxima were used for the detection and quantification of vesicles for each channel. Colocalization of vesicles was measured using the area of coincidence. To account for chromatic aberration, Z-stack images of TetraSpec microspheres (Thermofisher Scientific, T7279) were captured to detect and reject misaligned x, y and z-stack slices for each image. Differences between genotype and treatment for autophagosome and lysosome number, average size and colocalization in each layer were analyzed using a Two-way ANOVA with a Tukey multiple comparisons test (GraphPad prism, p < 0.05).

***Analysis of changes in protein expression in autophagy pathways***: A combination of reverse phase protein array and simple western analysis were used to quantify protein expression changes in the authophagy pathways. The reverse phase protein array technique allowed simultaneous, quantitative measurement of protein expression levels in a large number of samples using highly specific antibodies (Table 2) and small quantities of protein lysate for assessment of proteins of interest in a dot blot manner (Victorian Center for Functional Genomics, Peter MacCallum Cancer Institute) [52]. Simple western analysis for antibodies with multiple bands, specifically, LC3 were assessed using the JESS system (Protein Simple, Trendbio, Victoria, Australia), which runs small volumes of lysate within a capillary system according to size. Proteins of interest within the capillary are detected and quantified using specific primary antibodies and secondaries with fluorescent or chemiluminescent detection [53].

**Table 2.** Reverse phase protein array antibodies.

Antibody name	Host	Wb validation	Supplier
SRC	Rabbit IgG	Valid	Cell Signaling Technology, 2109
BCL2L1/Bcl-xL	Rabbit IgG	Valid	Cell Signaling Technology, 2762
AKT_P T308	Rabbit IgG	Valid	Cell Signaling Technology, 2965
MTOR_P S2448	Rabbit IgG	Valid	Cell Signaling Technology, 2971
MTOR	Rabbit IgG	Valid	Cell Signaling Technology, 2972
PTPN11/SHP-2_P Y542	Rabbit IgG	Valid	Cell Signaling Technology, 3751
RPS6KB/p70 S6 Kinase	Rabbit IgG	Valid	Cell Signaling Technology, 9202
AKT_P S473	Rabbit IgG	Valid	Cell Signaling Technology, 9271
AKT	Rabbit IgG	Valid	Cell Signaling Technology, 9272
PHB/prohibitin	Rabbit IgG	Valid	Santa Cruz Biotechnology, sc-28,259
EIF4EBP1/4E-BP1	Rabbit IgG	Valid	Cell Signaling Technology, 9452
EIF4EBP1/4E-BP1_P S65	Rabbit IgG	Valid	Cell Signaling Technology, 9456
EIF4EBP1/4E-BP1_P T37, TT46	Rabbit IgG	Valid	Cell Signaling Technology, 9459
ATM	Rabbit IgG	Valid	Cell Signaling Technology, 2873
TSC2/tuberin	Rabbit IgG	Caution	Cell Signaling Technology, 3612
p-MAPK14/p38 MAPK_P T180, Y182	Rabbit IgG	Valid	Cell Signaling Technology, 4631
RPS6/S6 ribosomal protein	Rabbit IgG	Valid	Cell Signaling Technology, 2217
CCNE1/cyclin E1	Rabbit IgG	Valid	Cell Signaling Technology, 20,808
PRKAA/AMPK-α	Rabbit IgG	Valid	Cell Signaling Technology, 8208
EPCAM	Rabbit IgG	Valid	Thermofisher Scientific, 710,524
TP53/p53	Mouse IgG1	Caution	Thermofisher Scientific, MA5-11,296
SQSTM1/p62	Mouse IgG2a	Valid	Novus Biologicals, H00008878-M01

For both reverse phase protein array and simple western analysis, retina and RPE from 13 month old WT- and APOE-mice (n = 5/genotype/treatment) were prepared after treatment from 5 months of age with metformin (0.4 g/kg/day) or trehalose (3 g/kg/day) provided in the drinking water. Additionally, to assess autophagy flux, experiments to block autophagy-lysosomal degradation were completed. At 5 months of age, WT- and APOE-mice were treated with metformin (0.4 g/kg/day) or trehalose (3 g/kg/day) provided in the drinking water, while sham controls received standard drinking water for 2 weeks. Using techniques published previously [34], WT- and APOE-retinae and RPE/choroid/sclera complex were collected from n = 5 animals/treatment group and incubated in culture media with or without 50 µM chloroquine (autophagy-lysosomal blocker; Sigma-Aldrich, C6628-25 G) for 24 h at 37°C in a humidified cell culture chamber with 5% CO_2_. The culture media consisted of Alpha minimum essential medium (Sigma-Aldrich, M4526-500ML), 1% L-glutamine (Gibco, 25,030,081), 1% penicillin-streptomycin (Gibco, 15,140–148), and 1% fetal bovine serum (Hyclone, SH30071.03). Retinae and RPE samples were isolated and lysed at room temperature in 30 µL of CBL1 buffer (7 M urea [Sigma-Aldrich/Merck, 108,487], 2 M thiourea [Sigma-Aldrich/Merck, T8656], 4% [w:v] CHAPS [Sigma-Aldrich/Merck, 220,201], 1% [w:v] dithiothreitol, 4 mM spermidine [Sigma-Aldrich/Merck, S2626], 2% [w:v] pharmalyte [Sigma-Aldrich/Merck, P1522], Roche cOmplete protease inhibitor [Sigma-Aldrich/Merck, 11,697,498,001]). Samples were spun at 7000 g for 2 min to pellet debris and the supernatant collected. Protein concentration was assessed at 595 nm using a Bradford assay 96-well plate assay (Coomassie [Bradford] Protein Assay Kit, [Thermo Fisher Scientific, 23,200]) and samples were diluted to 2 mg/mL for assay on the protein array and simple western detection system according to manufacturer’s recommendations. For assessing LC3 (Novus Biologicals, NB100-2220 as above) with the JESS system, samples were run with GAPDH as a protein loading control and detected using either fluorescence or chemiluminescence detection to improve sensitivity. Data for LC3 are expressed as a ratio of LC3-II:LC3-I. For the reverse phase protein array, traditional Western blot was completed on each antibody prior to use on the array, to ensure that only a single band of the correct molecular weight was detected [52]. Westen blot validation of antibodies that detect phosphorylated proteins, showing that these recognize a band correlating with the molecular weight of the phosphorylated protein are presented in Figure S2. Differences between genotype and treatment for each protein of interest were analyzed using a Two-way ANOVA for genotype and treatment, corrected for multiple comparisons by controlling the False Discovery Rate, using the Two-stage linear step-up procedure of Benjamini, Krieger and Yekutieli (GraphPad prism, p < 0.05).

## Supplementary Material

Supplemental Material
