# Supplementary material for: Treatments targeting autophagy ameliorate the age-related macular degeneration phenotype in mice lacking APOE (apolipoprotein E)
Source: Autophagy. 2022 Feb 23;18(10):2368–84. doi: 10.1080/15548627.2022.2034131 (PMC9542759; doi:10.1080/15548627.2022.2034131)
Supplement: Supplemental Material [file KAUP_A_2034131_SM1850.zip › supplementary/Vessey-ApoE-autophagy-paper+supp figs_070122-R2_kv.docx]

***
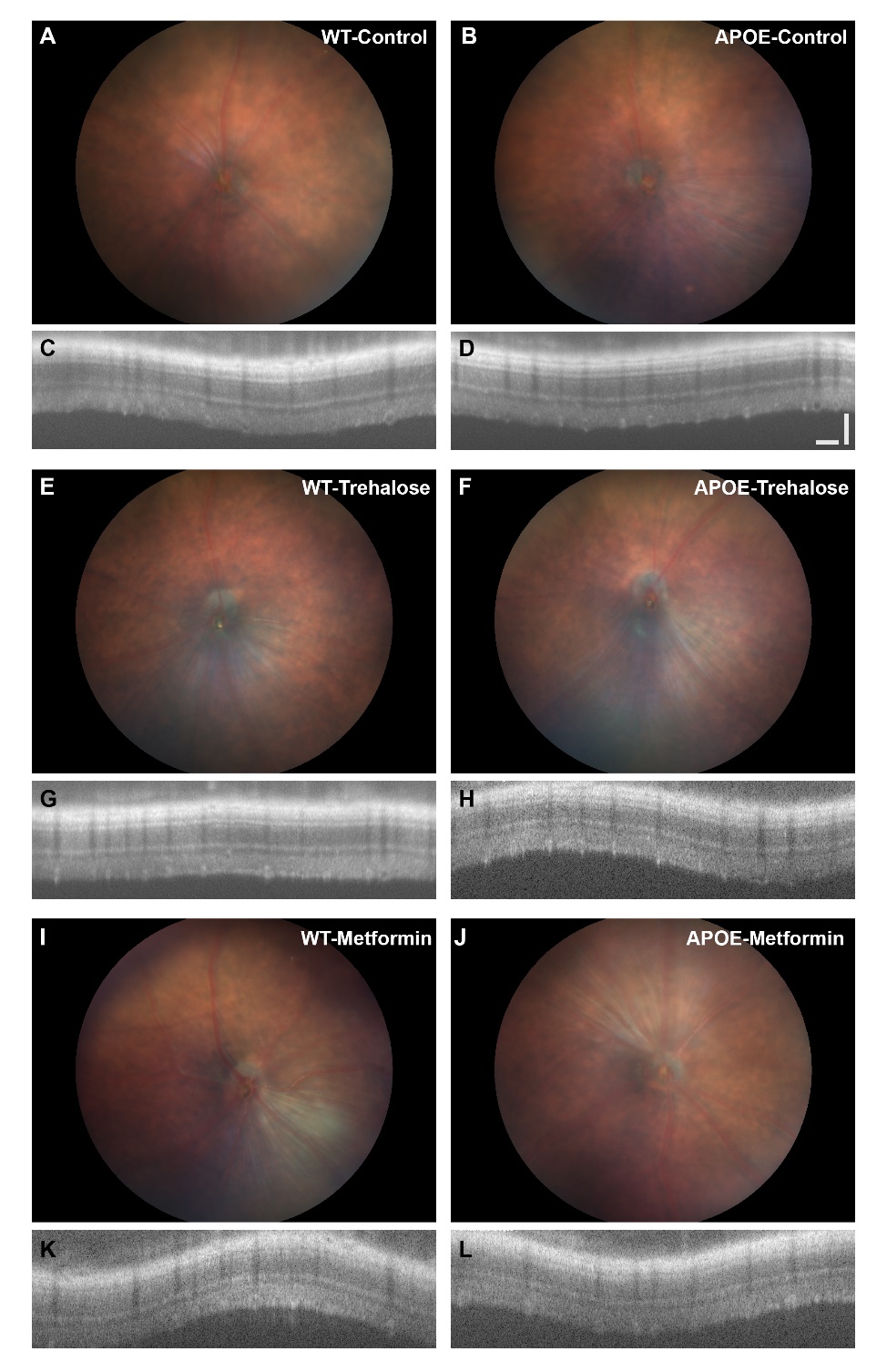
*Figure S1.** Fundus and optical coherence tomography for retinal layer thickness is not altered in APOE-mice or as a result of drug treatment. *In vivo* imaging was used to investigate ocular fundus appearance and retinal thickness in WT- and APOE-retinae at 13 months of age using spectral domain optical coherence tomography (OCT). (A-D) Fundus image from a (A) WT-control and (B) APOE-control mouse and their respective OCT images (C, WT; D, APOE) are presented. (E-H) Fundus image from a (E) WT-trehalose-treated and (F) APOE-trehalose-treated mouse and their respective OCT images (G, WT; H, APOE) are presented. (I-L) Fundus image from a (I) WT-metformin-treated and (J) APOE-metformin-treated mouse and their respective OCT images (K, WT; L, APOE) are presented. Retinal layer thickness was assessed by segmentation analysis of the OCT images and no effect of genotype or treatment was observed (Table 1). Scale X and Y: 50µm.


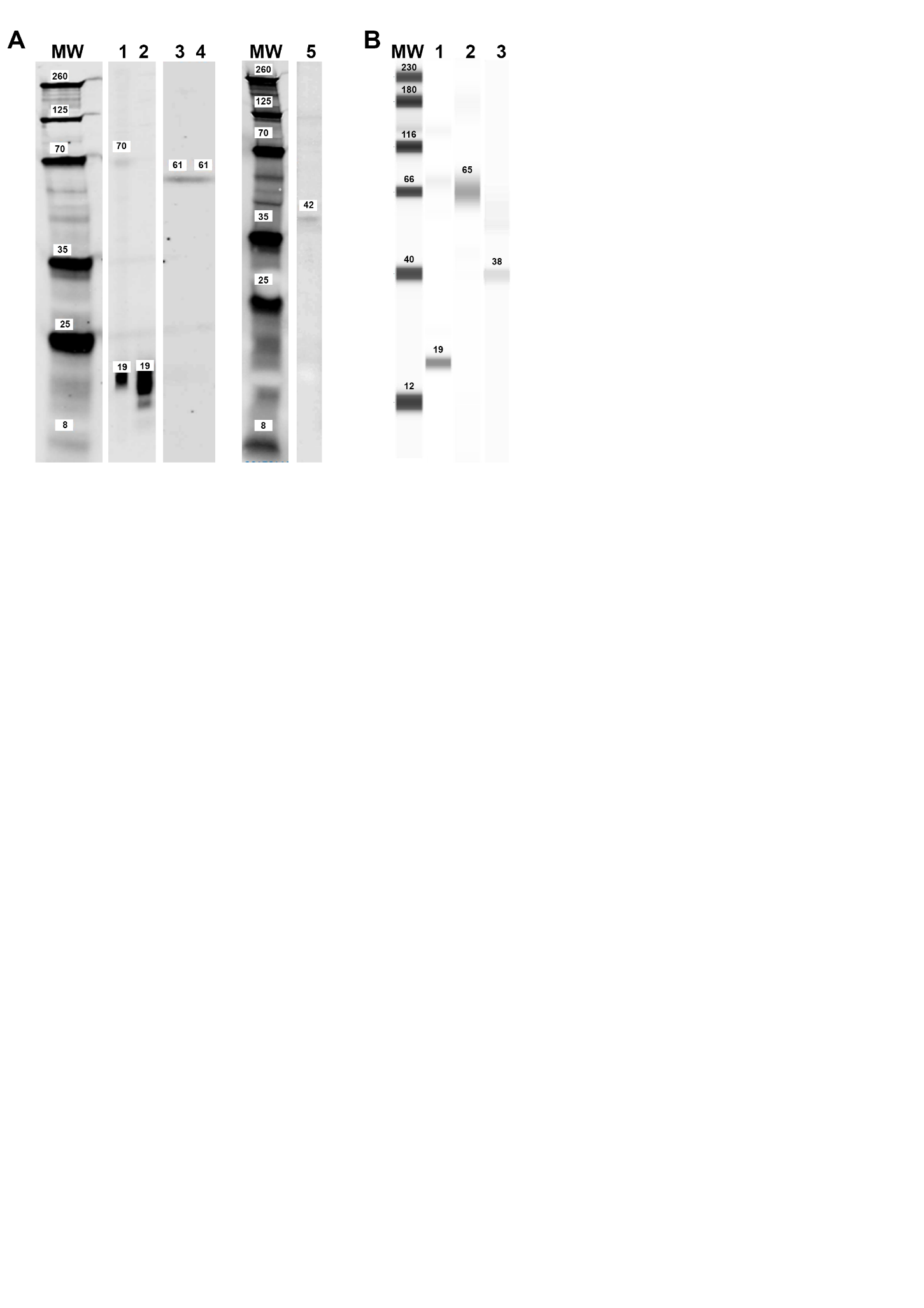


**Figure S2**. Western blot validation of antibodies targeting prosphorylated proteins used on the reverse phase protein array. All antibodies used on the reverse phase protein array were first tested using traditional western blot to ensure a specific band could be obtained. Validation data for the antibodies targeting the phosphorylated proteins are presented. (A) Protein from human cell lines, MBA MB 468 and OPM2 (samples heated to 70°C) of 0.6mg/mL were applied to a 4 to 12% gradient acrylamide-bis Tris gel (Bolt Bis-Tris 4-12%) along with a molecular weight marker (Chameleon Duo Pre-stained Protein Ladder; Licor, 928-60000). Proteins were separated by electrophoresis, transferred onto PVDF membranes and probed with 1:1000 dilution of primary antibody and 1:20,000 secondary, with chemiluminescent detection. Lanes presented: MW, Molecular weight, 1) p-EIF4EBP1 Ser65; 2) p-EIF4EBP1 Thr37, 46; 3) p-AKT Ser473; 4) p-AKT Thr308; 5) p-MAPK14/p38, show antibodies detect proteins of the expected molecular weight in human cell lines. (B) Retinal proteins from adult C57blk6J mice at 2mg/mL were assessed using Protein Simple Western analysis on a 12-230kDa gel/capillary system using 1:10 dilution of primary antibody and chemiluminescent detection. Lanes presented: MW, molecular weight, 1) p-EIF4EBP1 Thr37, 46; 2) p-AKT Thr308; 3) p-MAPK14/p38, show antibodies detect proteins of the expected molecular weight in mouse retina. The data sheet for these antibodies indicates cross reactivity in human, mouse and rat tissues.
